# Decarbonization and the Benefits of Tackling Climate Change

**DOI:** 10.3390/ijerph19137776

**Published:** 2022-06-24

**Authors:** María Mar Miralles-Quirós, José Luis Miralles-Quirós

**Affiliations:** Faculty of Economic and Business Sciences, University of Extremadura, 06006 Badajoz, Spain; miralles@unex.es

Since the industrial revolution, humans have increasingly influenced the Earth’s temperature and climate. Human practices such as deforestation, increased livestock farming, and the burning of fossil fuels such as coal, oil, and natural gas are causing huge amounts of gases to be emitted into the atmosphere. The accumulation of carbon dioxide, methane, nitrous oxide, and fluorinated gases from these activities is causing an increase in the greenhouse effect and, consequently, global warming.

Due to human activity, the temperature of our planet has increased by more than 1 °C in the space of 120 years. Human-induced global warming is currently increasing at a rate of 0.2 °C per decade. If we do not remedy the situation immediately, in less than 50 years, we will have reached a temperature increase of 2 °C above pre-industrial levels. This rise in global temperature is endangering life on Earth. The negative effects on the environment, human health, and well-being are already a reality. Moreover, if this human-induced phenomenon is left unchecked, the effects may be even more catastrophic in the near future.

We are already witnessing changes in our planet, such as extreme temperature peaks, with an increasing frequency of heat and cold waves; melting of glaciers and ice caps; flooding of cities and islands near the sea; increased air pollution in cities; increasingly devastating natural phenomena, such as hurricanes, torrential rains, and fires, which are increasing in both number and intensity; the migration of animal species to other habitats, modifying both the ecosystems of origin and the destination; the desertification of fertile areas; changes in agriculture and livestock farming, leading to food shortages, particularly affecting countries with fewer economic resources; and finally, the spread of diseases, plagues, and pandemics.

The 2021 report of the Intergovernmental Panel on Climate Change (IPCC) reflects that climate change is a global phenomenon affecting all regions of the planet and that centuries or millennia are needed to reverse all these adverse phenomena. The report also warns that the observed global warming is worse and faster than feared. Additionally, it states that around 2030, ten years earlier than estimated, the threshold of +1.5 °C could be reached, with “unprecedented” disaster risks for humanity. However, they also indicate that a substantial and sustained reduction in emissions of carbon dioxide (CO_2_) and other GHGs would limit climate change [1].

On the other hand, as the World Health Organization reports on its website, a healthy recovery of the planet is needed. This requires a carbon-free economy in which nature is restored and thrives. This will lead to a reduction in poverty; increased access to clean energy, clean water, and clean air; and a more reliable food supply, among other things. However, the whole of society must contribute to this fight.

In the fight against climate change, the work carried out by different institutions has been essential, especially the United Nations, which, through the development of numerous summits, has managed to bring many countries together with a common goal. The origin of these meetings dates to the 1994 Rio de Janeiro Convention, also known as the “Earth Summit”, where the United Nations Framework Convention on Climate Change (UNFCCC) was approved. The first Conference of the Parties (COP) was held in Berlin in 1995. Since then, the parties have met for two weeks, once a year. The venue rotates each year among the regional groups of the United Nations: Africa, Asia-Pacific, Western Europe, Latin America, the Caribbean, etc.

Among the 25 COPs held so far, the most important are COP3 in Kyoto, where the protocol of the same name was adopted, and where industrialized countries committed to limit and reduce GHG emissions in accordance with agreed individual targets; COP18 in 2012 in Doha, where a protocol was ratified; and most recently, COP21 in 2015, where the most important agreement to date in terms of climate change was approved: the Paris Agreement.

The Paris Agreement is a turning point in the common fight against climate change, as it is the first universal and legally binding agreement on climate change. As mentioned above, it was adopted at the Paris Climate Conference (COP21) on 12 December 2015 and subsequently ratified on 4 November 2016. It provides a lasting framework for global efforts for decades to come, and its main objective is to increase countriesʹ climate ambitions over time [2].

Since its entry into force, more and more countries, regions, cities, and companies are setting carbon neutrality targets. It can therefore be said that the Paris Agreement marks the beginning of a shift towards a low-carbon world. However, several studies have shown that there is still a long way to go, and a greater degree of ambition is needed. Rafteri et al. [3] placed the probability of achieving the Paris targets at 1%. From an economic point of view, Kahn et al. [4] argue that, if current policies remain unchanged, the global temperature increase will eventually have a significant impact on global GDP, which in per capita terms could reach 7.22% by 2050. Meanwhile, the 2021 report from IPCC suggests that the speed of implementation of the Paris Agreement is slow and insufficient to achieve the proposed goals. Specifically, emissions must be halved by 2030 and, above all, reach ‘net zero’ by 2050 to avoid the worst climate impacts. This means that warming of 1.5 °C or less compared to pre-industrial levels must be achieved [1].

The net zero emissions target implies that GHG emissions caused by human activity are equal to or in balance with those removed from the atmosphere. Achieving this progress requires drastic actions such as decarbonizing the economy and restoring forests. It is important to note that these drastic actions require the work not only of the public sector but also of the private sector. In this regard, each company and organization is called upon to set emission reduction targets following a science-based methodology. This is the only way to achieve substantial and significant emission reductions and, therefore, to reach the net zero target.

On 5 June 2020, World Environment Day, the High-Level Climate Action Champions of COP25, Gonzalo Muñoz, and of COP26, Nigel Topping, launched the “Race to Zero” campaign with this objective. (The figure of the High-Level Climate Action Champions originated in 2015, in the talks that led to the Paris Agreement, to designate specific individuals capable of leveraging and connecting the work of governments with the many voluntary actions of cities, regions, companies, and investors. Since then, each Conference of the Parties has had its own High-Level Champion). Specifically, it is a global campaign that seeks to mobilize non-state actors (regions, cities, businesses, investors, and educational institutions) to take rigorous and immediate action to drastically reduce global emissions by 2030 and achieve a zero-carbon world by 2050. However, the goal of this campaign is to mobilize and generate change towards a decarbonized economy that requires leadership from businesses, cities, regions, and investors to achieve a healthy, resilient, and carbon-free recovery that avoids future threats, creates decent jobs, and enables inclusive and sustainable growth.

This campaign is posed as a race against the clock towards net zero emissions based on the worrying reports issued by the IPCC. In addition, this campaign has given new impetus to the Climate Ambition Alliance, announced in 2019 in the context of COP25, which commits countries to be more ambitious but also promotes an active participation of the private sector to accelerate the transformation needed to achieve the Paris Agreement goals and stabilize the global temperature increase at 1.5 °C.

Finally, in 2022, the Race to Resilience has been created as a global campaign that is also supported by the United Nations to catalyze a step change in global climate resilience ambition, putting people and nature first in the quest for a resilient world where we not only survive climate shocks and stresses but thrive despite them. Its specific objectives are to (i) catalyze action by non-state actors that builds the resilience of 4 billion people in groups and communities vulnerable to climate risks by 2030, and to (ii) help the most vulnerable communities build resilience and adapt to the physical impacts of climate change in three types of areas: urban, rural, and coastal.

In this context of global action, this Special Issue, “Decarbonization and the Benefits of Tackling Climate Change” was proposed. These are cross-cutting issues that involve many areas of knowledge and must be present in different lines of research.

On the one hand, we must bear in mind that the health and lives of millions of people are at stake because of the risks associated with climate change. It is therefore necessary to provide innovative solutions to cover these risks or to deal with disasters that have already occurred. This requires engagement and collaboration among clinicians and public health researchers to address these public health challenges [5].

On the other hand, to achieve a decarbonized economy, business needs to be involved. Research can act as a lever in this regard by providing appropriate methodologies for measuring and reducing GHGs in different areas of business activity. In addition, it is also necessary to involve the financial sector in a unique way. It is essential to promote sustainable finance because the achievement of these objectives requires large amounts of financing [6,7].

Moreover, researchers should play an important role among non-state actors needed to resist this global problem. The prestige of the research provided by the scientific community will subsequently be reflected in the approval of public polices [8] and the improvement in voluntary social and business actions [9] that will enable net zero emissions to be achieved by 2050.

Finally, it is necessary to mention that *International Journal of Environmental Research and Public Health* is the best forum for discussion of discoveries and knowledge in these multidisciplinary fields.
